# Supplementary material for: Mobile Applications in Mood Disorders and Mental Health: Systematic Search in Apple App Store and Google Play Store and Review of the Literature
Source: Int J Environ Res Public Health. 2022 Feb 15;19(4):2186. doi: 10.3390/ijerph19042186 (PMC8871536; doi:10.3390/ijerph19042186)
Supplement: Supplementary file 1 [file ijerph-19-02186-s001.zip › ijerph-1564991-supplementary.pdf]

Table S1: Resulting apps with some available published evidence.

**Applications with published evidence**								
**Name**	**Developer**	**Developer profile**	**Type**	**Publications**	**Objective**	**Population**	**Type of data**	**URL**
7 Cups: Anxiety & Stress Chat	7 Cups of Tea	US-based team, also affiliated with a foundation	Chat & e-counselling	4 published articles [1-4]	Provide free active listening and paid online therapy	Various patient types, incl. stress, anxiety, depression, etc.	Written information (chat)	7cups.com
BetterHelp: Online Counseling & Therapy	BetterHelp - Therapy Made Easy	Professional developers, partnered with different mental health groups	E-counselling	1 published article [5]	Provide professional counselling, chat, and video messaging with therapist	Various patient types, incl. stress, anxiety, depression, etc.	Written information (chat, questionnaires), video call; chats are confidential	betterhelp.com
DBT Coach	Swasth Inc.	Psychologist as CMO	Cognitive behavioural therapy (CBT)	2 published articles [6,7]	Dairy, CBT/DBT (Dialectical Behavior Therapy) exercises, peer groups	Various patient types, incl. stress, anxiety, depression, etc.	Written information (tracker)	swasth.co/dbt-coach/
GGtude OCD Anxiety & Depression	Ggtude Ltd	Core team: Prof. Guy Doron, a clinical psychologist; and two founders with business background	CBT	6 published articles [8-13]	Applying CBT methods to break unhelpful thought patterns	OCD, anxiety, depression	Written information (mood tracker)	ggtude.com
Happify	Happify, Inc.	Team of scientists and experts	Tracker, meditation, exercises	3 published articles [14-16]	Changing negative thought patterns, tracker, and activities/videos	Various patient types, incl. stress, anxiety, depression, etc.	Written information (tracker)	happify.com
Joyable: An AbleTo Program	Joyable Inc	3 scientific advisors: Dr. Lisa McTeague, Dr. Rick Heimberg, Dr. Robert DeRubeis	CBT	1 published article [17]	2-month plan to deliver personalized CBT programs and support from coaches	Various patient types, incl. stress, anxiety, depression, etc.	Written information (chat, tracker)	joyable.com
MoodMission - Cope with Stress, Moods & Anxiety	MoodMission Pty Ltd	David Bakker, psychologist; Nikki Rickard, psych, prof.	Tracker, meditation, exercises	5 published articles [18-22]	Mood boosting activities, meditation, relaxation, exercise, affirmations, yoga, gratitude	Low mood, depression, stress, anxiety	None	moodmission.com
Talkspace Therapy & Counseling	Groop Internet Platform inc.	The CMO Neil Leibowitz has clinical background	E-counselling	5 published articles [23-27]	Provide professional counselling, chat, and video messaging with therapist	Various patient types, incl. stress, anxiety, depression, etc.	Written information (chat, questionnaires), video calls	talkspace.com

Table S2: Resulting apps with no available published evidence, but legitimate background.

**Applications without published evidence**							
**Name**	**Developer**	**Developer profile**	**Type**	**Objective**	**Population**	**Type of data**	**URL**
Aurum	Nirvaan Wellness	Unavailable	Therapy and programs	Self-education on CBT, etc. counselling with therapists	Various patient types, incl. stress, anxiety, depression, etc.	Written information (chat, questionnaires), video calls	aurumwellness.in
CBT Companion	Swasth Inc.	Psychologist as CMO	CBT	Companion for CBT, to be used with clinicians	Various patient types, incl. stress, anxiety, depression, etc.	Written information (tracker)	swasth.co/cbt-companion
Feel better	Media Local	Michael McConnell, has tech developer background	Tracker and various activities	CBT methods, mood tracking, change mood, chat bot, gratitude expression	Various patient types, incl. stress, anxiety, depression, etc.	Written information (tracker)	feelbetterapp.io
iFeel: Psicologos Online	ifeel	Amir Kaplan, Gabriele Murrone, tech background; holder of EU commission certificate of excellence	E-counselling	Psychological support and therapy with licensed psychologists in chat and video	Various patient types, incl. stress, anxiety, grief, low mood, panic, depression, etc.	Written information (chat), video calls	ifeelonline.com
InnerHour Self-Care Therapy - Anxiety & Depression	InnerHour	India-based team, psychologists on board	E-counselling, tracker, information	Provide advice on how to handle stress and depression and tracking progress	Anxiety, depression	Written information (chat, tracker), video calls	theinnerhour.com
LuzIA: Tu Coach Virtual	LuzIA	Enrique Martin, software engineer & Luz Rodriguez, engineer, and psychologist	AI chat	Conversational platform that uses AI; courses and instructional videos	Various patient types, incl. stress, anxiety, depression, etc.	Written information (chat)	luzia.chat
MediQuo Medical Chat	mediQuo chat médico	Some doctors on team	Chat/ e-counselling with doctors	24-hr medical chat with healthcare specialists	Various patient types, incl. stress, anxiety, depression, etc. (also available for other types of diseases and disorders)	Written information (chat), video calls	mediquo.com
Mind Ease	Mind Ease Labs Ltd	Team does not appear to have thorough scientific background	Meditation, tracker, combined with exercises	Stress and anxiety relieving exercises	Stress, anxiety	Written information (tracker)	mindease.io
My Possible Self	My Possible Self Ltd	Joanne Wilkinson heads team - some counselling background, associated with NHS Digital Apps	E-counselling & journal/ tracker	Personalised self-help tool, uses diary function and self-help modules, draws on established forms of therapy	Stress, anxiety, mild-moderate depression	Written information (chat, tracker), video calls	mypossibleself.com

		Library and Black Dog Institute					
Pocketcoach - Anxiety Helper	Pocketcoach	Some psychologists in team	CBT	Education on CBT, 6-week course including chat, audio, and exercise lessons	Stress, anxiety, panic	Written information (chat)	pocketcoach.co
Psonrie - Online psychologists	Psonrie	Spanish team, certified psychologists, support from Catalan government	E-counselling	Providing an online therapy platform	Various patients, incl. anxiety, stress, panic, depression, couples, self-esteem, etc.	Written information (chat), video calls	psonrie.com
Remente	Remente	Team from Sweden, seemingly no clinical background	Journal/ tracker and guides	Coaching and guides for habit forming	Stress, anxiety, depression	Written information (tracker/journal)	remente.com
Rootd	Simply Rooted Media	Unavailable	Courses, information, meditation, journal/tracker	CBT lessons, journal, mindfulness; panic attack and anxiety relief	Anxiety and panic attacks	Written information (tracker and short answers)	rootd.io
Serenmind	Serenmind	Team headed by Rosa Baños y Cristina Botella, psychology professors; EIT Health supported	CBT, chat, guide, tracker	Offers different programs to follow, mindfulness exercises and chat bot	Stress, anxiety, depression	Written information (tracker, chat)	serenmind.com
TherapyChat	TherapyChat	Team of certified psychologists from Madrid	E-counselling	Provide professional online therapy	Various patient types, incl. stress, anxiety, depression, etc.	Written information (chat), video calls	therapychat.com

Table S3: Resulting apps with no available published evidence and limited available information.

**Applications with limited available information**							
**Name**	**Developer**	**Developer profile**	**Type**	**Objective**	**Population**	**Type of data**	**URL**
Disminuya la depresión	Surf City Apps	Scripts written by certified hypnotherapists; little other information available	Hypnosis	Hypnosis to realise changes in mental health	Depression	None	es.surfcityapps.com
Friend Shoulder	TwoCats App	Unavailable	Anonymous chat	Anonymous chat to vent problems and receive advice	Various patient types, incl. stress, anxiety, etc	Written information (chat)	twocats.app
Meyo	Meyo tech	Unavailable	Information, digital content	Stress relief, healthy habit building and self-esteem building	Various patient types, incl. stress, anxiety, depression, and low self-esteem, etc.	None	meyo.io
Psychiatry Pro	BDR limited	Unavailable	Information & therapy elements	Diagnosis, information provision, psychotherapy, diary, substance abuse counselling, video counselling (TBD)	Various patient types, incl. stress, anxiety, depression, etc.	Written information (questionnaire, tracker)	No website available
Stop Panic & Anxiety Self-Help	Excel At Life	Associated with Dr. Monica Frank: Licensed psychologist	Journal, meditation, exercises	Learn about methods to manage panic and anxiety. Relaxation, mindfulness, and teaching audios. Mood log and analysis, cognitive diary, healthy goals	Anxiety and panic attacks	written info and diary	excelatlife.com
Termine con la ansiedad	Surf City Apps	Scripts written by certified hypnotherapists; little other information available	Hypnosis	Hypnosis to realise changes in mental health	Anxiety	None	es.surfcityapps.com
Yana: Tu acompañante emocional	YANA APP S.A.P.I. de C.V.	Andrea Campos	Chat bot	Routine building, chat bot	Various patient types, incl. stress, anxiety, etc	Written information (chat)	yana.com.mx

Table S4: Summary of all identified publications pertaining to the found mental health apps.

App	Intervention	Title	Study Type	Population	Outcomes
7 Cups: Anxiety & Stress Chat	Online platform of volunteer listener based emotional support, to complement treatment	Digital Peer-Support Platform (7Cups) as an Adjunct Treatment for Women With Postpartum Depression: Feasibility, Acceptability, and Preliminary Efficacy Study [1]	Non-randomised feasibility and acceptance study	Mothers experiencing postpartum depression (PPD) (N=19)	*Intent-to-treat analysis revealed that 7Cups recipients experienced significant decreases in Edinburgh Postnatal Depression Scale (EPDS) scores<br>*No significant difference in EPDS decrease over time was found between 7Cups and treatment as usual, but effect size favoured 7Cups
		Adaptation of a peer based online emotional support program as an adjunct to treatment for people with schizophrenia-spectrum disorders [2]	Descriptive study of platform adaptation	People with schizophrenia-spectrum disorders (N=10)	*168 listeners completed an online knowledge test<br>*Patients' attitudes towards the listeners were positive and they expected the platform to be usable and helpful
		Adjusting an Available Online Peer Support Platform in a Program to Supplement the Treatment of Perinatal Depression and Anxiety [3]	Descriptive study of platform development and adaptation	Women with PPD or anxiety (N=9)	*Patients found the platform usable and useful<br>*Their attitudes toward the trained listeners were positive.<br>*Patients noted a need for support outside the scheduled therapy time and believed that freely available online emotional support could help meet this need.
		Online emotional support delivered by trained volunteers: users' satisfaction and their perception of the service compared to psychotherapy [4]	User satisfaction study (online survey)	Convenience sample of platform users (N=1139)	*Findings showed high user satisfaction with the support<br>*Findings suggest that receiving support from volunteers makes users feel that the support is more genuine
BetterHelp: Online Counseling & Therapy	Multimodal digital psychotherapy platform	Effectiveness of a Multimodal Digital Psychotherapy Platform for Adult Depression: A Naturalistic Feasibility Study [5]	Non-randomised feasibility and effectiveness study	Convenience sample of platform users (N=318)	*Depression symptom severity was significantly reduced after the use of the multimodal digital psychotherapy intervention<br>*Individuals without prior traditional psychotherapy experience revealed increased improvement after intervention<br>*No significant dose-response effect was found
DBT (Dialectical Behaviour Therapy) Coach	Smartphone app designed to enhance generalization of DBT skill among individuals with borderline personality disorder (BPD)	The DBT Coach mobile application as an adjunct to treatment for suicidal and self-injuring individuals with borderline personality disorder: A preliminary evaluation and challenges to client utilization. [6]	Pilot feasibility, acceptability, usability study	Individuals with BPD and recent history of attempted suicide and/or no suicidal self-injury (NSSI) (N=16)	*Results indicate good acceptability and usability of the DBT Coach<br>*Analyses indicated the DBT Coach reduced subjective distress and urges to self-harm following app use.<br>*App use was not related to any treatment outcomes, except for reductions in NSSI
		A Pilot Study of the DBT Coach: An Interactive Mobile Phone Application for Individuals With Borderline Personality Disorder and Substance Use Disorder [7]	Pilot effectiveness and feasibility study	Individuals with BPD and comorbid substance use disorders (SUD) (N=22)	*Participants used the DBT Coach an average of nearly 15 times and gave high ratings of helpfulness and usability<br>*Indications that both emotion intensity and urges to use substances significantly decreased within each coaching session<br>*Over the trial period, participants reported a decrease in depression and general distress

GGtude OCD Anxiety & Depression	Platform designed to challenge maladaptive beliefs using short, interactive, touchscreen-based interventions to reduce psychological symptoms	Reaching reliable change using short, daily, cognitive training exercises delivered on a mobile application: The case of Relationship Obsessive Compulsive Disorder (ROCD) symptoms and cognitions in a subclinical cohort [8]	Randomised controlled study	University students exhibiting ROCD features and symptoms (N=50; 25 immediate use, iApp; 25 delayed use, dApp)	*Repeated measure MANOVA showed a significant Group × Time interaction and a significant main effect for time<br>*These interactions indicated greater decrease in ROCD symptoms, OCD beliefs and social anxiety symptoms, as well as a greater increase in self-esteem in the iApp group compared to dApp group<br>*Reliable Change Index indicated reliable change on ROCD symptoms for a significant portion of participants
		Cognitive Behavioral Training Using a Mobile Application Reduces Body Image-Related Symptoms in High-Risk Female University Students [9]	Randomised controlled study	Female university students at high-risk of developing body image disorders (BIDs) (N=50; 25 immediate use, iApp; 25 delayed use, dApp)	*Repeated measure Analyses of Variance showed a Group × Time interaction on BDD symptoms indicating medium effect size reductions in the iApp group compared to dApp group<br>*post-intervention means for body dysmorphic disorder symptoms were under the cut-off for extreme symptoms in both groups<br>*No significant Group × Time interaction was detected pertaining to eating disorder symptoms
		Can Brief, Daily Training Using a Mobile App Help Change Maladaptive Beliefs? Crossover Randomized Controlled Trial [10]	Crossover Randomized Controlled Trial	Nonclinical university students (N=97; 51 immediate use, iApp; 46 delayed use, dApp)	*App use was associated with medium-large effect size reductions in both iApp and dApp groups<br>*Analyses revealed significant treatment × repeated measures interactions on maladaptive beliefs, several obsessive-compulsive disorder (OCD) symptom measures, and self-esteem
		Can self-esteem be improved using short daily training on mobile applications? Examining real world data of GG Self-esteem users [11]	Observational study of app use	App users (N=5,320 at baseline; N=1,034 second assessment; N=165 third assessment)	*Significant increases in self-esteem ratings were found across all three time points<br>*Increased mood ratings were only found at second assessment, compared to baseline
		Assisting relapse prevention in OCD using a novel mobile app-based intervention: A case report [12]	Case study	26 y/o female patient (N=1) with severe cleaning OCD symptoms	*The patient completed 47 levels dedicated to OCD-relevant maladaptive beliefs<br>*The OBQ-20 and OCI-R scores decreased from pre- to post-GGOCD
		Building resilience to body image triggers using brief cognitive training on a mobile application [13]	Randomized controlled study	Nonclinical sample of women (N=90; 48 intervention; 42 control)	*Relative to control, participants who used the application demonstrated increased resiliency and reduced BID symptoms<br>*These effects were medium-to-large and were maintained at 1-month follow-up
Happify	Digital intervention	Effect of Brief Biofeedback via a Smartphone App on Stress Recovery [14]	Randomized Experimental Study	Participants (N=140) recovering in one of	*Participants using the game Breather on the Happify app had significantly lower levels of salivary alpha amylase during recovery than participants in the other conditions

	platform addressing mental health by drawing on theoretical approaches			three ways from laboratory stressor	
		The Effects of a Digital Well-Being Intervention on Patients With Chronic Conditions [15]	Observational study	App users (N=821) with (N=450) and without (N=371) chronic condition	*Both, users with and without a chronic condition experienced equivalent improvement in well-being, despite users with a chronic condition having significantly lower subjective well-being at baseline<br>*Completing more activities and doing so over increasingly longer periods produced improved well-being scores
		The Impact of a Digital Intervention (Happify) on Loneliness During COVID-19 [16]	Qualitative Focus Group	Participants recruited from a pilot randomised controlled trial (N=11)	*There was considerable heterogeneity in participants' experiences with loneliness, but participants commonly referred to negative self-perceptions as a cause or consequence of loneliness<br>*Regarding the intervention, participants showed evidence of adopting skills used to address loneliness, particularly mindfulness and gratitude, and using these to shift toward more active coping strategies following the intervention
Joyable: An AbleTo Program	Open-access, coach-supported CBT-based intervention	Evaluation of an open-access CBT-based Internet program for social anxiety: Patterns of use, retention, and outcomes. [17]	Observational study	App users (N=3,384)	*Full adherence to the program was achieved by 16% of participants<br>*Social anxiety symptoms were significantly reduced for participants that engaged in the program<br>*Exposures and coach contact were significant predictors of retention and outcome
MoodMission - Cope with Stress, Moods & Anxiety	CBT strategy app serving as adjunct to therapeutic interventions delivered by trained health professionals, in the form of real-time, momentary responses to user-reported low moods and anxiety	A randomized controlled trial of three smartphone apps for enhancing public mental health [18]	Randomized controlled study	Volunteers (N=226) randomized to control or one of three mental health apps	*Compared to control all intervention groups experienced increases in mental wellbeing<br>*MoodKit and MoodMission groups experienced decreases in depression, and no groups experienced effects on anxiety<br>*Analysis showed that increasing coping self-efficacy was the main underlying process contributing to positive effects all 3 tested apps
		Effectiveness of Using Mental Health Mobile Apps as Digital Antidepressants for Reducing Anxiety and Depression [19]	Protocol for a Multiple Baseline Across-Individuals Design	Participants with mild-to-moderate anxiety and/or depression (N=50)	n/a<br>*Evaluation of 5 different apps
		Development and Pilot Evaluation of Smartphone-Delivered Cognitive Behavior Therapy Strategies for Mood- and Anxiety-Related Problems: MoodMission [20]	Descriptive study of app development and pilot	Nonclinical volunteers (N=44), who rated app usability	*MoodMission was rated significantly higher than standardized health app norms on most of the domains, including Entertainment, Customization, Target Group, Visual Appeal, Quality of Information, Quantity of Information, Credibility of Source, and Overall Rating

			usability evaluation		
		Engagement with a cognitive behavioural therapy mobile phone app predicts changes in mental health and wellbeing: MoodMission [21]	Observational study	App users (N=617)	*App engagement ratings predicted increases in mental wellbeing<br>*Mediation analyses revealed that there were indirect effects of app engagement on depression, anxiety, and mental wellbeing via the mediator of coping self-efficacy
		Engagement with smartphone-delivered behavioural activation interventions: a study of the MoodMission smartphone application [22]	Observational study of app engagement	App users (N=238)	*The average number of app-based activities completed was 5.46<br>*Average self-reported engagement level was low to moderate<br>*Higher levels of engagement significantly predicted more positive activity appraisal
Talkspace Therapy & Counselling	Telemedicine platform used by independently practicing, licensed therapists	Two-way messaging therapy for depression and anxiety: longitudinal response trajectories [23]	Longitudinal observational study	Outpatient sample presenting with a chief complaint of anxiety or depression (N = 10,718)	*Subpopulations Recovery and Acute Recovery had Generalized Anxiety Disorder (GAD-7) and Patient Health Questionnaire (PHQ-9) remission outcomes<br>*Subpopulations Depression and Anxiety Improvement showed amelioration of symptoms
		Just in time crisis response: suicide alert system for telemedicine psychotherapy settings [24]	Descriptive study of algorithm development	Training dataset of therapy transcripts for 1,864 patients	*The final Natural Language Processing model identified risk-related content from non-risk content with good accuracy
		Message Delivery for the Treatment of Posttraumatic Stress Disorder [25]	Longitudinal Observational Study	Outpatients diagnosed with PTSD (N=475)	*Growth mixture modelling identified 4 trajectories of PTSD symptoms: moderate improvement (41.4%), high symptoms (41.4%), chronic symptoms (12.9%), and acute improvement (4.3%)
		A Study of Asynchronous Mobile-Enabled SMS Text Psychotherapy [26]	Pilot feasibility study	Adults seeking text therapy treatment for variety of disorders (N=57)	*Results indicate evidence for the effectiveness of the intervention<br>*46% of participants experienced clinically significant symptom remission<br>*High levels of satisfaction with text therapy were reported<br>*Cost-effectiveness analyses suggest that text therapy is 42.2% the cost of traditional services and offers much reduced wait times
		Analyzing Digital Evidence From a Telemental Health Platform to Assess Complex Psychological Responses to the COVID-19 Pandemic: Content Analysis of Text Messages [27]	Context analysis	Text messages concerning COVID-19	*Significant increase in the incidence of COVID-19–related intake anxiety symptoms<br>*No significant differences in the incidence of intake depression symptoms

References

1. Baumel, A.; Tinkelman, A.; Mathur, N.; Kane, J. M. Digital Peer-Support Platform (7Cups) as an Adjunct Treatment for Women With Postpartum Depression: Feasibility, Acceptability, and Preliminary Efficacy Study. *JMIR MHealth UHealth* **2018**, *6* (2), e38. https://doi.org/10.2196/mhealth.9482.
2. Baumel, A.; Correll, C. U.; Birnbaum, M. Adaptation of a Peer Based Online Emotional Support Program as an Adjunct to Treatment for People with Schizophrenia-Spectrum Disorders. *Internet Interv.* **2016**, *4*, 35–42. https://doi.org/10.1016/j.invent.2016.03.003.
3. Baumel, A.; Schueller, S. M. Adjusting an Available Online Peer Support Platform in a Program to Supplement the Treatment of Perinatal Depression and Anxiety. *JMIR Ment. Health* **2016**, *3* (1), e11. https://doi.org/10.2196/mental.5335.
4. Baumel, A. Online Emotional Support Delivered by Trained Volunteers: Users' Satisfaction and Their Perception of the Service Compared to Psychotherapy. *J Ment Health* **2015**, *24* (5), 313–320. https://doi.org/10.3109/09638237.2015.1079308.
5. Marcelle, E. T.; Nolting, L.; Hinshaw, S. P.; Aguilera, A. Effectiveness of a Multimodal Digital Psychotherapy Platform for Adult Depression: A Naturalistic Feasibility Study. *JMIR MHealth UHealth* **2019**, *7* (1), e10948. https://doi.org/10.2196/10948.
6. Rizvi, S. L.; Hughes, C. D.; Thomas, M. C. The DBT Coach Mobile Application as an Adjunct to Treatment for Suicidal and Self-Injuring Individuals with Borderline Personality Disorder: A Preliminary Evaluation and Challenges to Client Utilization. *Psychol. Serv.* **2016**, *13* (4), 380–388. https://doi.org/10.1037/ser0000100.
7. Rizvi, S. L.; Dimeff, L. A.; Skutch, J.; Carroll, D.; Linehan, M. M. A Pilot Study of the DBT Coach: An Interactive Mobile Phone Application for Individuals With Borderline Personality Disorder and Substance Use Disorder. *Behav. Ther.* **2011**, *42* (4), 589–600. https://doi.org/10.1016/j.beth.2011.01.003.
8. Cerea, S.; Ghisi, M.; Bottesi, G.; Carraro, E.; Broggio, D.; Doron, G. Reaching Reliable Change Using Short, Daily, Cognitive Training Exercises Delivered on a Mobile Application: The Case of Relationship Obsessive Compulsive Disorder (ROCD) Symptoms and Cognitions in a Subclinical Cohort. *J. Affect. Disord.* **2020**, *276*, 775–787. https://doi.org/10.1016/j.jad.2020.07.043.
9. Cerea, S.; Ghisi, M.; Bottesi, G.; Manoli, T.; Carraro, E.; Doron, G. Cognitive Behavioral Training Using a Mobile Application Reduces Body Image-Related Symptoms in High-Risk Female University Students: A Randomized Controlled Study. *Behav. Ther.* **2021**, *52* (1), 170–182. https://doi.org/10.1016/j.beth.2020.04.002.
10. Roncero, M.; Belloch, A.; Doron, G. Can Brief, Daily Training Using a Mobile App Help Change Maladaptive Beliefs? Crossover Randomized Controlled Trial. *JMIR MHealth UHealth* **2019**, *7* (2), e11443. https://doi.org/10.2196/11443.
11. Giraldo-O'Meara, M.; Doron, G. Can Self-esteem Be Improved Using Short Daily Training on Mobile Applications? Examining Real World Data of GG Self-esteem Users. *Clin. Psychol.* **2020**, cp.12219. https://doi.org/10.1111/cp.12219.
12. Pascual-Vera, B.; Roncero, M.; Doron, G.; Belloch, A. Assisting Relapse Prevention in OCD Using a Novel Mobile App–Based Intervention: A Case Report. *Bull. Menninger Clin.* **2018**, *82* (4), 390–406. https://doi.org/10.1521/bumc.2018.82.4.390.
13. Aboody, D.; Siev, J.; Doron, G. Building Resilience to Body Image Triggers Using Brief Cognitive Training on a Mobile Application: A Randomized Controlled Trial. *Behav. Res. Ther.* **2020**, *134*, 103723. https://doi.org/10.1016/j.brat.2020.103723.
14. Hunter, J. F.; Olah, M. S.; Williams, A. L.; Parks, A. C.; Pressman, S. D. Effect of Brief Biofeedback via a Smartphone App on Stress Recovery: Randomized Experimental Study. *JMIR Serious Games* **2019**, *7* (4), e15974. https://doi.org/10.2196/15974.
15. Parks, A. C.; Williams, A. L.; Kackloudis, G. M.; Stafford, J. L.; Boucher, E. M.; Honomichl, R. D. The Effects of a Digital Well-Being Intervention on Patients With Chronic Conditions: Observational Study. *J. Med. Internet Res.* **2020**, *22* (1), e16211. https://doi.org/10.2196/16211.

16. Boucher, E. M.; McNaughton, E. C.; Harake, N.; Stafford, J. L.; Parks, A. C. The Impact of a Digital Intervention (Happify) on Loneliness During COVID-19: Qualitative Focus Group. *JMIR Ment. Health* **2021**, *8* (2), e26617. https://doi.org/10.2196/26617.
17. Dryman, M. T.; McTeague, L. M.; Olino, T. M.; Heimberg, R. G. Evaluation of an Open-Access CBT-Based Internet Program for Social Anxiety: Patterns of Use, Retention, and Outcomes. *J. Consult. Clin. Psychol.* **2017**, *85* (10), 988–999. https://doi.org/10.1037/ccp0000232.
18. Bakker, D.; Kazantzis, N.; Rickwood, D.; Rickard, N. A Randomized Controlled Trial of Three Smartphone Apps for Enhancing Public Mental Health. *Behav. Res. Ther.* **2018**, *109*, 75–83. https://doi.org/10.1016/j.brat.2018.08.003.
19. Marshall, J. M.; Dunstan, D. A.; Bartik, W. Effectiveness of Using Mental Health Mobile Apps as Digital Antidepressants for Reducing Anxiety and Depression: Protocol for a Multiple Baseline Across-Individuals Design. *JMIR Res. Protoc.* **2020**, *9* (7), e17159. https://doi.org/10.2196/17159.
20. Bakker, D.; Kazantzis, N.; Rickwood, D.; Rickard, N. Development and Pilot Evaluation of Smartphone-Delivered Cognitive Behavior Therapy Strategies for Mood- and Anxiety-Related Problems: MoodMission. *Cogn. Behav. Pract.* **2018**, *25* (4), 496–514. https://doi.org/10.1016/j.cbpra.2018.07.002.
21. Bakker, D.; Rickard, N. Engagement with a Cognitive Behavioural Therapy Mobile Phone App Predicts Changes in Mental Health and Wellbeing: MoodMission. *Aust. Psychol.* **2019**, *54* (4), 245–260. https://doi.org/10.1111/ap.12383.
22. Aizenstros, A.; Bakker, D.; Hofmann, S. G.; Curtiss, J.; Kazantzis, N. Engagement with Smartphone-Delivered Behavioural Activation Interventions: A Study of the MoodMission Smartphone Application. *Behav. Cogn. Psychother.* **2020**, 1–13. https://doi.org/10.1017/S1352465820000922.
23. Hull, T. D.; Malgaroli, M.; Connolly, P. S.; Feuerstein, S.; Simon, N. M. Two-Way Messaging Therapy for Depression and Anxiety: Longitudinal Response Trajectories. *BMC Psychiatry* **2020**, *20* (1), 297. https://doi.org/10.1186/s12888-020-02721-x.
24. Bantilan, N.; Malgaroli, M.; Ray, B.; Hull, T. D. Just in Time Crisis Response: Suicide Alert System for Telemedicine Psychotherapy Settings. *Psychother. Res.* **2021**, *31* (3), 289–299. https://doi.org/10.1080/10503307.2020.1781952.
25. Malgaroli, M.; Hull, T. D.; Wiltsey Stirman, S.; Resick, P. Message Delivery for the Treatment of Posttraumatic Stress Disorder: Longitudinal Observational Study of Symptom Trajectories. *J. Med. Internet Res.* **2020**, *22* (4), e15587. https://doi.org/10.2196/15587.
26. Hull, T. D.; Mahan, K. A Study of Asynchronous Mobile-Enabled SMS Text Psychotherapy. *Telemed. J. E-Health Off. J. Am. Telemed. Assoc.* **2017**, *23* (3), 240–247. https://doi.org/10.1089/tmj.2016.0114.
27. Hull, T. D.; Levine, J.; Bantilan, N.; Desai, A. N.; Majumder, M. S. Analyzing Digital Evidence From a Telemental Health Platform to Assess Complex Psychological Responses to the COVID-19 Pandemic: Content Analysis of Text Messages. *JMIR Form. Res.* **2021**, *5* (2), e26190. https://doi.org/10.2196/26190.
